# Augmentation of cognitive function in epilepsy

**DOI:** 10.3389/fnsys.2014.00147

**Published:** 2014-08-14

**Authors:** Thomas B. DeMarse, Paul R. Carney

**Affiliations:** ^1^J. Crayton Pruitt Family Department of Biomedical Engineering, University of FloridaGainesville, FL, USA; ^2^Department of Pediatrics, University of FloridaGainesville, FL, USA

**Keywords:** non-invasive brain stimulation, tDCS, memory, cognition, epilepsy

Epilepsy is one of the most common neurological disorders in humans afflicting more than 1% of the population and 65 million people worldwide (England et al., 2012). The most common form of acquired epilepsy is temporal lobe epilepsy (TLE), and over 30% of patients with TLE have seizures that are refractory to commonly used anticonvulsant drugs (Bauer and Burr, 2001). Mesial temporal lobe sclerosis (MTS) is the most common pathological abnormality in TLE (Bronen et al., 1997). The histopathological hallmarks of hippocampal sclerosis include segmental loss of pyramidal neurons, granule cell dispersion, and reactive gliosis (Sutula et al., 1989). Indeed, changes in the integrity of hippocampus and surrounding hippocampal white matter is postulated to influence overall temporal lobe network connectivity, hippocampus efficiency, seizures (Cadotte et al., 2009), and memory function (Eichenbaum et al., 2007, 2012). Indeed, animal and human studies show that abnormalities in the hippocampus and its white matter inputs and outputs are correlated with the severity of memory dysfunction (Christidi et al., 2011).

## Temporal lobe connectivity in TLE: filling gaps in knowledge

Patterned inputs to the hippocampus from mediobasal cortical regions and entrorhinal cortex are hypothesized to support critical memory functions of recollection (controlled, deliberate recall) and familiarity (automatic, item-based memory), respectively (Eichenbaum and Lipton, 2008; Eichenbaum et al., 2012; Dixon et al., 2014). An integrated theory of parahippocampal (PHc), perirhinal (PRc), entrorhinal (ERc), and hippocampal (HC) functioning [the ‘Binding of Items and Context [BIC] Model (Diana et al., 2007)], suggests that these structures form an integrated circuit that supports recollection and familiarity. The PRc is proposed to be important in encoding and retrieving items (e.g., objects, words, and ideas), whereas the PHc is responsible for representing spatial, temporal, and semantic context. The HC supports memory for episodes by integrating these inputs and binding the item-based contextual information together as a unique event in space and time. In this view, the formation of new memories depends upon the integrated series of inputs from PRc and PHc components of the parahippocampal gyrus and their respective targets in the ERc and hippocampus. Despite extensive animal work, it is unknown in humans whether selective damage to these areas or their interconnections that produce subtypes of memory impairment might differentially respond to different types of memory training. Although some data exists on the efficacy of memory rehabilitation programs, little is known about the neural basis of individualized rehabilitation responses from a mechanistic perspective (Wagner, 2011).

## Implications for memory rehabilitation

Understanding individual differences in morphological and connectional components of medial temporal lobe injury in TLE can lead to identification of subtypes of memory impairment, and thus help identify clinically important targets for memory augmentation. Our hypothesis is that the subtypes of memory impairment that result will preferentially respond to specific memory interventions, a notion that is also being addressed in the aphasia treatment literature (Kim et al., 2011). To this end, an emerging method for treating neurologically-induced memory impairment is non-invasive brain stimulation (NIBS), including transcranial direct current stimulation (tDCS) and transcranial magnetic stimulation (TMS). Both tDCS and TMS are safe for use in human subjects (Nitsche et al., 2003b), and have been used widely to test hypothesis about causal links between specific brain structures supporting cognition and memory (Dayan et al., 2013; Hummel, 2014). Indeed, several studies support the use of NIBS techniques as tools for enhancing cognitive function in normal subjects and as therapeutic agents for individuals with psychiatric and neurologic disorders (Hummel and Cohen, 2006; Miniussi et al., 2008). NIBS consist of applying a weak (0.5–2.0 mA in tDCS) direct current through the scalp and skull. Depending on the polarity of the current during stimulation, NIBS may increase or decrease the rate of neuronal firing by modulating the resting membrane potentials (Creutzfeldt et al., 1962; Bindman et al., 1964; Liebetanz et al., 2002; Nitsche et al., 2003a; Zaghi et al., 2010). Although these studies are preliminary, they do provide reassuring proof-of-principle that the stimulated brain region is part of a critical circuit for performing the task under investigation.

The application of brain stimulation in combination with specific memory rehabilitation methods (Stringer and Small, 2011) has been put forth as a strategy to compensate for basic defects in TLE-related memory processing (Miatton et al., 2011; Sankar et al., 2012; Suthana et al., 2012; Fell et al., 2013; Hariz et al., 2013; Hartikainen et al., 2014; Suthana and Fried, 2014). These studies demonstrate that electrical neuromodulation of specific deep structures within the medial temporal lobe may have persistent benefits in memory function.

NIBS has been shown to significantly decrease seizures in individuals with treatment-resistant epilepsy (Fregni et al., 2006; Nitsche and Paulus, 2009; San-Juan et al., 2011; Varga et al., 2011; Yook et al., 2011; Auvichayapat et al., 2013; Parazzini et al., 2014). Whether NIBS techniques can also improve memory function in TLE is an area of much interest. To this end, recent reports suggest that NIBS may augment cognition in a wide array of neurologic and psychiatric disorders, including schizophrenia (Minzenberg and Carter, 2012), Alzheimer's disease (Boggio et al., 2006), depression (Brunoni et al., 2012), and post-stroke recovery (Floel, 2014). Although the underlying mechanism that produces the cognitive deficits associated with epilepsy may differ from those that produce similar deficits in other disorders, the mechanism that enables tDCS' therapeutic effect appears to transcend individual disease. These results strongly suggest that tDCS may represent an excellent potential new treatment modality for epilepsy. Therefore, future studies on the possible effects of tDCS in TLE are highly warranted. There are however, a number of significant issues that must be addressed for tDCS to become practical as a treatment for TLE.

## Future directions

While NIBS has been shown to be relatively safe, currently there is surprisingly little known about the specific mechanisms underlying the therapeutic effects (Reato et al., 2013). Nevertheless, various postulates have been put forward such as N-methyl-D-aspartate receptor mediated long and short-term potentiation modulation (Liebetanz et al., 2002; Nitsche et al., 2004; Thickbroom and Mastaglia, 2009). Studies aimed at defining the dose for NIBS techniques in space and in time, as well as determining the safe stimulation intensity parameters and electrode positions, are now critical to propel this field forward. Finally, with regard to tDCS, it was initially believed to primarily affect cortical regions directly beneath the electrode. However, there are now a number of reports based on results from computer modeling suggesting that the current during tDCS may in fact reach deeper areas, such as the hippocampus (Sadleir et al., 2010; Parazzini et al., 2012). In order to systemically reach the hippocampus and surrounding structures at therapeutic levels, computer modeling will be needed and will likely play an increasingly important role in the design of electrode montages that can consistently reach these areas in the future. Fortunately, a number of groups now use computer modeling to gain a better understanding of where current is flowing during NIBS as well as methods to guide or focus current (Datta et al., 2009; Bai et al., 2013; Dmochowski et al., 2013; Edwards et al., 2013). While NIBS techniques offer the capability to modulate large or diverse areas of the brain, it is still an open question as to what extent electrical neuromodulation in one brain area may affect adjacent or more distant areas and mechanism of action. However, recent efforts are beginning to explore these many complex issues directly (Keeser et al., 2011; Polania et al., 2011; Lamy et al., 2012; Polania et al., 2012; Park et al., 2013; Hampstead et al., 2014; Notturno et al., 2014).

Future advancements in current methodologies for NIBS may provide substantial improvements during focal delivery of stimulation to the temporal lobe for memory augmentation. Also, improvements in multi-modal non-invasive techniques such as fMRI or MEG, may be able to detect neural signatures reflective of NIBS related neurophysiological changes within the hippocampus and surrounding structures that result in memory enhancement. Through the combined use of NIBS and multiunit local field potential recordings in combination with non-invasive measurements such as EEG and fMRI studies we may be able to optimize detection and determine the precise neuronal correlates of NIBS related behavioral changes. Other training techniques such as neurofeedback may also allow patients the ability to modulate electrical stimulation oscillatory activity in order to achieve improvements in memory.

In summary, it will become increasingly important for future studies to build upon and elucidate the mechanism of action used in NIBS enhancement of memory. The location, parameters, and phase of delivery of NIBS may need to vary amongst individuals. Hence, systematic comparisons and consistent methodologies across studies will likely contribute to a solid understanding of NIBS and its effects on learning and memory. Resolution of these issues may be crucial as to whether NIBS based therapeutics will advance toward a useful treatment for patients with TLE related memory problems.

### Conflict of interest statement

The authors declare that the research was conducted in the absence of any commercial or financial relationships that could be construed as a potential conflict of interest.
